# The use of online support by people with intellectual disabilities living independently during COVID‐19

**DOI:** 10.1111/jir.12770

**Published:** 2020-08-24

**Authors:** M. Zaagsma, K.M. Volkers, E.A.K. Swart, A.P. Schippers, G. Van Hove

**Affiliations:** ^1^ Philadelphia Care Foundation Amersfoort The Netherlands; ^2^ Department of Medical Humanities, Amsterdam Public Health research institute Amsterdam UMC, VU University Medical Center Amsterdam The Netherlands; ^3^ Department of Special Needs Education Ghent University Ghent Belgium; ^4^ Disability Studies in the Netherlands De Meern The Netherlands

**Keywords:** corona, COVID‐19, intellectual disabilities, online support, remote support, services

## Abstract

**Background:**

During the COVID‐19 outbreak, service providers in the Netherlands had to switch towards providing remote support for people with intellectual disabilities living independently. This study aims to provide insight into the use of online support during the outbreak.

**Methods:**

We analysed quantitative data on planned and unplanned contacts between the online support service DigiContact and its service users.

**Results:**

The results indicate that the COVID‐19 outbreak and the related containment measures had a strong impact on online support use, specifically on the unplanned use of online support.

**Conclusion:**

Offering online support as a standard component of services for independently living people with intellectual disability enables service providers to be flexible and responsive towards fluctuations in both support needs and onsite support availability during a social crisis such as COVID‐19.

## Background

The global outbreak of the COVID‐19 virus and the restrictive measures imposed by governments aimed at containing its spread have a strong impact on the provision of social care and support services for people with disabilities around the world (Armitage and Nellums 2020; European Association of Service Providers for Persons with Disabilities 2020). It seems likely that also people with intellectual disabilities (IDs) have been (or still are) at risk of experiencing a discontinuation of support to some extent. For example, in the Netherlands, while residential care services for people with ID continued, visits from friends and family were mostly prohibited. Services such as day activity centres and meeting centres, as well as some sheltered workshops, were put on hold for several months (Dutch Association for Healthcare Providers for People with Disabilities 2020; Woittiez *et al*. 2020). Although at the time of writing this paper (June 2020), restrictive measures are being lifted, it remains to be seen whether relapses will occur causing measures to be reinstated.

While in the Netherlands, considerable (media) attention was paid to the impact of restrictive measures on people in residential services and on their families, the impact on people with ID who live independently has been relatively underexposed. With day activity centres and some sheltered workshops being put on hold, people risked temporarily losing their daytime occupation that not only provided them with structure in their day, but also implied the loss of an important vehicle to community participation and social inclusion (Brooks *et al*. 2020; Lysaght *et al*. 2016; Simplican *et al*. 2015). In addition, service providers had to switch towards offering support as much as possible remotely, for example, through online video calls, to safeguard the health of both support staff and service users (Dutch Association for Healthcare Providers for People with Disabilities 2020; European Association of Service Providers for Persons with Disabilities 2020). For people with ID, such a sudden change may result in an experience of being thrown back to one's own resources, especially when family and friends cannot provide (more) support due to the containment measures (Courtenay and Perera 2020; de Vries *et al*. 2020).

Although it is still early to evaluate efforts to provide remote support during the COVID‐19 outbreak, the specific case of the Dutch online support service DigiContact presents us with the opportunity to get insights into the use of online support by people with ID living independently. DigiContact was developed and implemented in 2014 by the service provider organisation Philadelphia Care Foundation (Vijfhuizen and Volkers 2016). People with ID can contact a team of specially trained support workers 24/7, either planned or unplanned (whenever they feel the need). Contact can be realised through specially developed videoconferencing techniques (using a PC, laptop, tablet or smartphone) or through a regular phone connection. Depending on a person's support needs, online support is usually combined with onsite support. As DigiContact was already operational before the COVID‐19 outbreak, the capacity for online contacts could relatively quickly be scaled up by adding resources such as necessary equipment and bringing in onsite support staff, who could no longer provide onsite support, to increase the size of the online support staff team. In addition, a team of nurses was installed that could be consulted through DigiContact for COVID‐19‐related medical questions.

This study aims to contribute to the knowledge on the usefulness of offering remote, online support to independently living people with ID during a time of crisis, when regular onsite services are not or less available. With this aim, we explored the use of DigiContact support during the first weeks of the COVID‐19 pandemic. In this paper, we focus on the following question: how does the (planned and unplanned) use of online support by people with ID living independently evolve during the first weeks of the COVID‐19 pandemic in the Netherlands?

## Methods

A retrospective, descriptive research design was employed in which quantitative data on support contacts between DigiContact support staff and independently living people with ID were employed. The Medical Ethics Review Committee of VU University Medical Center (FWA00017598) confirmed that the Dutch Medical Research Involving Human Subjects Act (WMO) did not apply to this study and that official approval by the committee was therefore not required. The privacy officer of the involved service organisation reviewed and approved the research plan and procedure.

Before the COVID‐19 outbreak, the number of independently living persons with ID connected to DigiContact fluctuated around 700. During the pandemic, 282 additional persons were connected at the initiative of the service provider, to create a safety net for them in the event that many onsite support workers would fall ill and/or regular support (from a distance) could not be continued. Being connected to DigiContact means that several technical and administrative actions have been completed that enable a person to contact the service. However, being connected does not necessarily imply that someone actually has contacts with the service. Persons who actually have contacts with the service are from now on referred to with the term service users.

We used data on online support contacts from the service provider's administrational systems to explore the use of DigiContact. As our focus was on the use of online support during the first months of the COVID‐19 pandemic, we used data on contacts during the first 20 weeks of 2020. To enable a comparison with the use of DigiContact support during a similar period without a pandemic outbreak, we also included data on contacts during the first 20 weeks of 2019. All data were anonymised before being handed over to the first author for analysis: a unique identification code was allocated to each service user, which enabled us to identify contacts belonging to the same service user. The data set included the following information for each support contact: date, start time, a service user identification number (recoded for anonymisation) and whether a contact was unplanned or planned.

**Figure 1:**
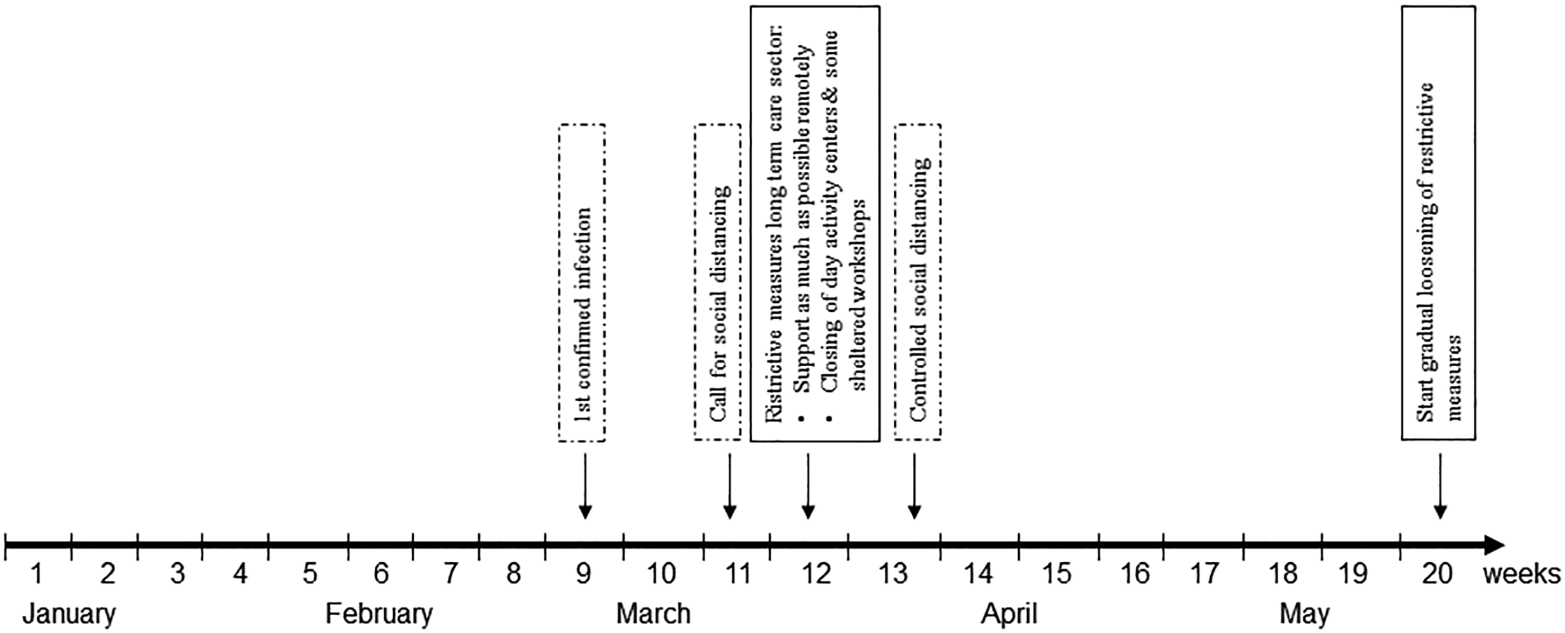
Timeline of Dutch COVID‐19 containment measures impacting service provision to people with intellectual disability living independently.

Analysis was performed using spss version 23 for Windows and Microsoft Excel 2016. First, descriptive statistics were run regarding the number of service users and (unplanned and planned) contacts per day during the period with active COVID‐19 containment measures (weeks 12–20 of 2020, see Fig. 1) and two reference periods: (1) the weeks prior to the period with active containment measures (weeks 1–11 of 2020) and (2) the first 20 weeks of 2019. To get a more detailed overview of how online support use evolved over time, we also calculated the number of unplanned and planned contacts per day for each week. Second, we compared the amount of unplanned and planned contacts per day per service user during the COVID‐19 period and both reference periods. Given the non‐normal distribution of the data, non‐parametric two‐tailed Wilcoxon signed‐rank tests were used to test for differences between periods. The significance level was set at 2.5% to adjust for multiple testing, as we had two reference groups. The findings were presented to two senior members of the DigiContact team, and possible interpretations were discussed.

## Results

**Figure 2:**
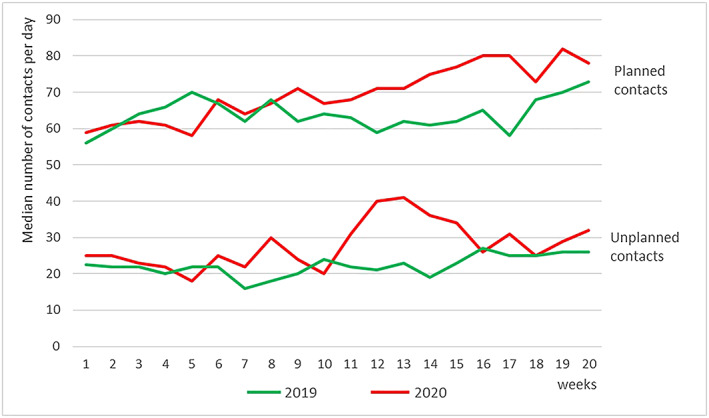
The number of planned and unplanned support contacts per day, over the first 20 weeks of 2020 and 2019. [Colour figure can be viewed at wileyonlinelibrary.com]

A total of 648 service users had at least one contact during the first 20 weeks of 2019 and/or the first 20 weeks of 2020. Of these service users, 32 had been newly connected to DigiContact during (and due to) COVID‐19 and therefore only had contacts during the pandemic. Table 1 presents the median scores and interquartile range of the number of contacts per day between DigiContact and its users: it indicates that the service dealt with a higher number of contacts per day during COVID‐19 than during the two reference periods. Figure 2 presents the patterns in the number of contacts per day during the first 20 weeks of 2020 and 2019 and therefore gives a detailed view on how the amount of contacts evolved over the weeks. The 2020 patterns were more or less comparable with those of 2019 up to week 10/11. In weeks 11/12 (2020), the number of unplanned contacts per day considerably increased, and after which, it slowly decreased again from week 13 and reached a level comparable with before COVID‐19 in week 16. Although the number of planned contacts per day also increased during COVID‐19, this increase was more gradual and continued longer than the unplanned contacts.

**Table 1:** Median scores and interquartile ranges of the amount of support contacts per day, during COVID‐19 and two reference periods

Period	Service users	Unplanned contacts/day	Planned contacts/day	All contacts/day
	*n*	Mdn (IQR)	Mdn (IQR)	Mdn (IQR)
Old service users	616			
COVID‐19^*^	466	32.00 (24.00–40.00)	74.00 (70.00–79.00)	106.00 (96.00–116.00)
Ref. 2020^†^	445	23.00 (19.00–28.00)	63.00 (57.00–70.00)	88.00 (78.00–97.00)
Ref. 2019^‡^	435	22.00 (18.00–26.00)	64.00 (57.00–72.00)	86.00 (77.00–97.00)
New service users				
COVID‐19^*^	32	0.00 (0.00–1.00)	1.00 (0.00–2.00)	2.00 (1.00–3.00)

Service users are people with at least one contact with DigiContact during a specific period. Old service users were already connected before COVID‐19 started. New service users were connected during the COVID‐19 period.

^*^ COVID‐19 period: weeks 12–20, 2020.

^†^ Reference period: weeks 1–11, 2020.

^‡^ Reference period: weeks 1–20, 2019.

IQR, interquartile range.

Table 2 presents the median and interquartile range of the amount of contacts per day per service user, of the group of service users who had already been using the service before the pandemic (*N* = 616). The amount of unplanned contacts per day per service user was significantly higher during COVID‐19 than during the first 11 weeks of 2020 (*z* = −4.602, *P* = .000), as well as than during the first 20 weeks of 2019 (*z* = −5.328, *P* = .000). Although the amount of planned contacts per day per service user during COVID‐19 did not differ significantly from the first 11 weeks of 2020 (*z* = −1.776, *P* = .076), it was significantly higher than during the first 20 weeks of 2019 (*z* = −3.689, *P* = .000).

**Table 2:** Median scores and interquartile ranges for the number of support contacts per day per service user, during COVID‐19 and two reference periods

	COVID‐19^*^	Ref. 2020^†^		Ref. 2019^‡^	
	Mdn (IQR)	Mdn (IQR)	COVID‐19^*^ vs. Ref. 2020^†^	Mdn (IQR)	COVID‐19^*^ vs. Ref. 2019^‡^
Unplanned contacts	.016 (.000–.032)	.000 (.000–.027)	*z = −4.602, P = .000*	.007 (.000–.022)	*z = −5.328, P = .000*
Planned contacts	.048 (.000–.143)	.040 (.000–.133)	*z = −1.776, P = .076*	.036 (.000–.128)	*z = −3.689, P = .000*
Total contacts	.079 (.000–.144)	.067 (.000–.160)	*z = −3.626, P = .000*	.058 (.000–.144)	*z = −4.832, P = .000*

Wilcoxon signed rank tests (*N* = 616) were used to test for differences between number of (unplanned and planned) contacts during COVID‐19 period and both reference periods.

^*^ COVID‐19 period: weeks 12–20, 2020.

^†^ Reference period: weeks 1–11, 2020.

^‡^ Reference period: weeks 1–20, 2019.

IQR, interquartile range.

## Discussion

The findings show that the use of the online support service DigiContact by independently living people with ID increased during the first weeks of the COVID‐19 pandemic, as people had more (unplanned) contacts with the service than before the outbreak. In addition, there was a small group of people who were newly connected to the service during (and often due to) COVID‐19 and who started to use the service and thereby also contributed to the increase in online support use. The instalment of containment measures in week 12 was accompanied by a substantial increase in the unplanned use of online support (without appointment), which lasted for a few weeks. The planned use of online support (with appointment) also seemed to gradually increase over a longer time than the unplanned contacts, but without the group of new service users, this increase was not statistically significant compared with the first 11 weeks of 2020.

These findings indicate that the COVID‐19 outbreak and related restrictive measures had quite an impact on the use of online support. A possible explanation for the sudden, substantial and temporary increase in unplanned online support use is that people were considerably worried and experienced a high level of anxiety especially during the first weeks of the crisis, causing more people to contact the service (more often). Several authors have pointed out that people with ID (like people without ID) are likely to experience high levels of stress and frustrations during the COVID‐19 pandemic and that measures posing restrictions on their usual activities and contacts with other people further contribute to this (Brooks *et al*. 2020; Courtenay and Perera 2020; de Vries *et al*. 2020). In a previous study, we found that DigiContact was often used to ventilate worries and frustrations with an aim to relieve oneself of them (Zaagsma *et al*. 2019). A lack of accessible information on the virus and the containment measures (Courtenay and Perera 2020) may also have contributed to the increase in unplanned online support use as, especially during the first weeks, people with ID were left with questions that caused them to contact the service. The finding that the unplanned online support use decreased after a few weeks may have been caused by the initial feelings of worry and anxiety subsiding. This resonates with the concept of homeostatic effects on subjective well‐being: that each person (irrespective of any disabilities) has a certain set point of well‐being and a homeostatic control makes us return to this set point automatically after a deviation (Cummins 2017; Cummins *et al*. 2011). However, it is also possible that the unplanned use of online support did not decrease because the need for support diminished, but because service users experienced that unplanned contacts were not (sufficiently) effective in helping them and stopped to initiate these contacts. For example, it is possible that service users experienced that DigiContact staff could not give them the answers and reassurance regarding COVID‐19‐related questions or worries that they had hoped for.

The (un)availability of services and the influence that support staff can exert on the use of services may also have played a role in the patterns of online support use. During the consternation of the first few weeks of COVID‐19, onsite support professionals and people with ID had to find and get used to a new strategy to be in contact with each other, which leads to a temporary interruption of (or at least an irregularity in) onsite support contacts. This may have resulted in an initial increase in people seeking (more) help online, as DigiContact may have been part of this new strategy of providing services. Service users who had unplanned contacts during the first weeks of COVID‐19 were offered the possibility of having a standing appointment with the service (planned contacts). This may have contributed to the decrease in unplanned contacts and the slow increase of planned contacts. The fact that arranging more online support capacity took some time may have played a role in the finding that planned online support use started to increase slower and later than the unplanned contacts.

Although this study provides some useful insights into how the use of online support evolves during the first weeks of the COVID‐19 pandemic, we acknowledge that these results are preliminary. For the interpretation of our findings, it would have been valuable to also include data on the use of other services in general and contacts with onsite support professionals specifically. Furthermore, this study focused on the first weeks of the COVID‐19 pandemic only. Although these first few weeks constitute an interesting period because of the sudden need to change the way support was provided, it would also be useful to look at the impact during a longer time frame.

The findings of this study suggest that by including online support as an addition to regular onsite support for people with ID living independently, service provider organisations are able to increase their responsiveness towards changes in the demand of support by service users and to compensate (at least partially) for changes in onsite support availability during a crisis like COVID‐19. This ties in with a finding of a previous study, in which we found that online support could move and adapt to fluctuations in support needs more easily compared with regular onsite services (Zaagsma *et al*. 2020). However, it should be noted that the online support service in this study had already been operational for several years when the COVID‐19 pandemic started, which enabled a fast response. Setting up a similar service quickly in reaction to COVID‐19 would, in all likelihood, have been very difficult, if not impossible.

The relevance of offering online services extends beyond the current COVID‐19 pandemic. In the recent decades, austerity measures in many countries have led to a reduction in onsite support availability and eligibility (Malli *et al*. 2018), and in this context, service provider organisations often see remote support (e.g. online) as a way to organise services more efficiently (Niemeijer *et al*. 2010; Tassé *et al*. 2020) and enhance support availability for those who need it. However, it is important to point out that previous findings indicate that online support should not be seen as a cost‐saving substitute for all onsite support contacts, but rather as a valuable addition to onsite support (Zaagsma *et al*. 2020).

In conclusion, this paper shows the value of incorporating online support as a standard component of a broader system of professional services for people with ID who live independently during a societal crisis, such as the current COVID‐19 virus outbreak. By already being operational when the outbreak happened, DigiContact enabled the system of services to be more flexible and responsive towards shifting levels of demand for professional support during the hectic first weeks that were possibly due to fluctuations in the support needs of people with ID and in the availability of onsite support.

## Acknowledgements

Our sincere gratitude goes out to everybody who helped us by making the data accessible, by preparing the data for analysis and/or by sharing their ideas and views on our findings. A special thank you goes out to Judith Rijnhart and Mark van de Wiel for their statistical advice and support.

## Conflict of Interest

This study was conducted as part of the dissertation research project of the first author. The authors declare that no restrictions were imposed during any phase of this study nor on the publication of the research data.

## Source of Funding

The study was funded by the service provider Philadelphia Care Foundation, Amersfoort, the Netherlands.
